# Exosomes from Drug-Resistant Breast Cancer Cells Transmit Chemoresistance by a Horizontal Transfer of MicroRNAs

**DOI:** 10.1371/journal.pone.0095240

**Published:** 2014-04-16

**Authors:** Wei-xian Chen, Xue-min Liu, Meng-meng Lv, Lin Chen, Jian-hua Zhao, Shan-liang Zhong, Ming-hua Ji, Qing Hu, Zhou Luo, Jian-zhong Wu, Jin-hai Tang

**Affiliations:** 1 The Fourth Clinical School of Nanjing Medical University, Nanjing, Jiangsu, China; 2 Department of General Surgery, Nanjing Medical University Affiliated Cancer Hospital, Cancer Institute of Jiangsu Province, Nanjing, Jiangsu, China; 3 Graduate School, Xuzhou Medical College, Xuzhou, Jiangsu, China; 4 Center of Clinical Laboratory, Nanjing Medical University Affiliated Cancer Hospital, Cancer Institute of Jiangsu Province, Nanjing, Jiangsu, China; 5 Department of Radiation Oncology, Nanjing Medical University Affiliated Cancer Hospital, Cancer Institute of Jiangsu Province, Nanjing, Jiangsu, China; 6 Center of Clinical Oncology, Nanjing Medical University Affiliated Cancer Hospital, Cancer Institute of Jiangsu Province, Nanjing, Jiangsu, China; University of South Alabama, United States of America

## Abstract

Adriamycin and docetaxel are two agents commonly used in treatment of breast cancer, but their efficacy is often limited by the emergence of chemoresistance. Recent studies indicate that exosomes act as vehicles for exchange of genetic cargo between heterogeneous populations of tumor cells, engendering a transmitted drug resistance for cancer development and progression. However, the specific contribution of breast cancer-derived exosomes is poorly understood. Here we reinforced other's report that human breast cancer cell line MCF-7/S could acquire increased survival potential from its resistant variants MCF-7/Adr and MCF-7/Doc. Additionally, exosomes of the latter, A/exo and D/exo, significantly modulated the cell cycle distribution and drug-induced apoptosis with respect to S/exo. Exosomes pre-treated with RNase were unable to regulate cell cycle and apoptosis resistance, suggesting an RNA-dependent manner. Microarray and polymerase chain reaction for the miRNA expression profiles of A/exo, D/exo, and S/exo demonstrated that they loaded selective miRNA patterns. Following A/exo and D/exo transfer to recipient MCF-7/S, the same miRNAs were significantly increased in acquired cells. Target gene prediction and pathway analysis showed the involvement of *miR-100*, *miR-222*, and *miR-30a* in pathways implicated in cancer pathogenesis, membrane vesiculation and therapy failure. Furthermore, D/exo co-culture assays and miRNA mimics transfection experiments indicated that *miR-222*-rich D/exo could alter target gene expression in MCF-7/S. Our results suggest that drug-resistant breast cancer cells may spread resistance capacity to sensitive ones by releasing exosomes and that such effects could be partly attributed to the intercellular transfer of specific miRNAs.

## Introduction

Breast cancer (BCa) is the most common tumor in women around world. Although significant advances have been made in chemotherapy, drug resistance remains a major clinical obstacle to successful treatment and leads to poor prognosis for the patients [1]. BCa cells effectively evade chemotherapy by a number of different processes and strategies. Among them, exosomes acting as mediators of intercellular communication are increasingly researched [2]. Exosomes are small vesicles 50 to 100 nm in diameter that are released upon fusion of multivesicular bodies with plasma membranes from diverse cell types [3]. Once thought to be “cell debris”, they are now considered important regulators in tumor biology including angiogenesis, invasiveness, evasion of immune surveillance and metastasis [4]–[7]. Exosomes contain mRNAs, microRNAs (miRNA), and proteins that could be transferred to target cells inducing epigenetic changes [8]–[10]. Moreover, accumulating evidence suggests that, in tumor patients, miRNAs circulate in body fluids in a highly stable and cell-free form, probably due to their incorporation in exosomes, allowing their use as novel diagnostic and prognostic markers [11].

It is generally recognized that tumors comprise a heterogeneous population of cells with marked differences in their chemo-susceptibility [12]. While the majority of malignant cells are attacked and ultimately eliminated after toxic insult, a minor population of cells, named as drug-resistant cells, would be undamaged and could spread resistance traits to residual cells during the course of treatment [13]. Recently, we established from human BCa cell line MCF-7 two variants that respectively display insensitivity properties to adriamycin (adr) and docetaxel (doc), such as altered cell cycle distribution, expression of MDR1, MRP1 and BCRP resistance-associated proteins, and reduction of apoptosis-promoting Bax [14]. These sublines (MCF-7/Adr and MCF-7/Doc), along with the sensitive parental one (MCF-7/S), could therefore be used as the models for investigating mechanisms of chemotherapy failure [15].

Previous studies showed that drug-resistant tumor cells are an abundant source of exosomes that may serve as paracrine modulators by a horizontal transfer of genetic cargo [16]–[18]. The aim of the present study was to evaluate whether exosomes derived from MCF-7/Adr and MCF-7/Doc may transmit chemoresistance to MCF-7/S by regulating cell cycle and apoptosis.

## Materials and Methods

### Cell culture

Human BCa cell line MCF-7 was purchased from the Cell Bank of the Chinese Academy of Sciences (Shanghai, China). Drug-resistant sublines, MCF-7/Adr and MCF-7/Doc, were successfully established from the parental MCF-7/S in our laboratory as recently described [14]. MCF-7/S was cultured synchronously, unexposed to adr or doc, as a control for all experiments. MCF-7/Adr and MCF-7/Doc were maintained in drug-free medium for two weeks before subsequent experiments to avoid the influence of toxic insult. For co-culture assays and uptake assays, MCF-7/S expressing green fluorescent protein (GFP-S) was prepared [19]. Briefly, MCF-7/S in logarithmic phase was transfected with the lentivirus encoding green fluorescent protein when a confluence of 50–60% was reached, according to the manufacturer's protocol (GenePharma, China). After a 72-hour incubation, the cells were examined under a fluorescence microscope (Carl Zeiss, Germany). Transfection was highly efficient (>99%), thus obviating the need to sort positive cells. Expression remained stable under prolonged culture.

All cell lines were grown in Dulbecco's modified Eagle's medium (DMEM) high glucose (HyClone, USA) supplemented with 10% fetal bovine serum (FBS), 100 U/ml penicillin, and 100 µg/ml streptomycin at 37°C under a water-saturated 95% air-5% CO_2_ atmosphere. For all studies, exosome-depleted FBS was prepared by centrifuging FBS at 100,000 g overnight to spin down any preexisting vesicular content.

### Exosome isolation

Exosomes were harvested from supernatants of MCF-7/Adr, MCF-7/Doc and MCF-7/S cultured in DMEM with 10% exosome-depleted FBS by differential centrifugation and ultracentrifugation as previously described [9]. The exosomes were designated as A/exo, D/exo and S/exo for simplicity. Briefly, cell culture media were sequentially centrifuged at 300 g for 10 min, 2,000 g for 15 min, and 12,000 g for 30 min to remove floating cells and cellular debris. These supernatants were passed through a 0.22 µm filter. The filtrates were further ultracentrifuged by an Avanti J-30I (Beckman Coulter, USA) at 100,000 g for 2 h at 4°C to collect exosomal pellets, which were then washed by resuspending in phosphate-buffered saline (PBS) and ultracentrifuged at 100,000 g for another 2 h at 4°C. The final pellets, comprised of exosomes, were used immediately or resuspended in 100 µl PBS and stored at −80°C. In selected experiments, A/exo, D/exo and S/exo were treated with 5 U/ml RNase (Ambion, USA) for 3 h at 37°C (RNase A/exo, RNase D/exo and RNase S/exo), the reaction was stopped by addition of 10 U/ml RNase inhibitor (Ambion, USA) followed by ultracentrifugation [8], [20]. The efficacy of RNase treatment was evaluated after RNA extraction (discussed below) by spectrophotometer analysis of total RNA. In addition, RNA obtained from RNase-treated and -untreated exosomes was analyzed on 0.6% agarose gel to show the complete degradation of RNA by RNA treatment.

### Exosome identification

Exosome morphology and size were determined by transmission electron microscopy as previously described [16]. In short, 15 µl exosome samples were dripped onto parafilm and covered with a 300 mesh copper grid. After 45 min, the copper mesh was washed with PBS, fixed in 3% glutaraldehyde, washed with double-distilled water and finally contrasted in 2% uranyl acetate. Images were obtained using a JEM-1010 electron microscope (JEOL, Japan) at an accelerating voltage of 80 kV. Cytofluorimetric analysis (BD FACSCalibur, USA) for the detection of surface molecules was performed [8], using a fluorescein isothiocyanate (FITC)-conjugated antibody directed to CD44 (DakoCytomation, Denmark). FITC mouse non-immune isotypic IgG (DakoCytomation, Denmark) was used as a control.

Western blot analysis of exosome-related proteins was carried out. Cellular and exosomal proteins were extracted using the RIPA lysis buffer (Biouniquer Technology, China) and Total Exosome RNA and Protein Isolation Kit (Invitrogen, USA) according to the manufacturer's instructions, respectively. The protein concentration was measured on a Nanodrop 2000 spectrophotometry (Thermo Scientific, USA). Equal amounts of proteins were subjected to electrophoresis on 10% SDS-polyacrylamide gels and transferred to polyvinylidene difluoride membranes (Sigma, Germany). After blocking with 5% skim milk powder in Tris-buffered saline (TBS)-Tween for 2 h, the membranes were incubated with mouse monoclonal anti-Tsg101 (1∶200; Santa Cruz, USA) and rabbit polyclonal anti-calnexin (1∶200; Santa Cruz, USA) followed by horseradish peroxidase-linked secondary antibodies. The secondary antibodies were goat anti-mouse IgG (1∶3000; Kangwei, China) and goat anti-rabbit IgG (1∶3000; Kangwei, China). β-actin (Sigma, Germany) was used as an internal loading control to normalize the expression pattern of each sample. After washing, bound proteins were detected using the ECL Plus Kit (Millipore, USA) with Image Lab Software (Bio-Rad, USA).

### Uptake assays

Exosomes were labeled with the red fluorescent dye PKH26 (Sigma-Aldrich, USA) according to the manufacturer's recommendation [9]. Briefly, while the isolated exosomes from 200 ml culture media were resuspended in 1 ml Diluent C, 4 µl PKH26 was diluted in another 1 ml Diluent C. Then, these two solutions were mixed gently for 5 min, after which 5 ml 1% bovine serum albumin was added to bind the excess dye. The mixture was subsequently ultracentrifuged at 100,000 g for 2 h at 4°C, washed with PBS by ultracentrifugation and finally resuspended in complete medium. As the negative control, exosomes without PKH26 staining were prepared. Incorporation of exosomes into GFP-S was visualized by fluorescence microscopy (Carl Zeiss, Germany) after incubation with 20 µg/ml PKH26-labeled A/exo or D/exo for 30 min at 37°C. Continued absorption was determined by flow cytometry analysis (BD FACSCalibur, USA) of the percentage of PKH26-positive GFP-S. Twenty four hours later, they were observed under a confocal laser scanning microscope LSM710 (Carl Zeiss, Germany). While excitation was achieved with the 488 nm laser line for GFP and the 543 nm laser line for PKH26, the emission light passes through a 530/30 nm and 573/26 nm band filter, respectively.

### Co-culture assays

As in a previous work [21], to investigate potential transmission of chemoresistance, either MCF-7/Adr or MCF-7/Doc or MCF-7/S cells (5×10^4^ cells per well) were mixed with GFP-S at equal proportions in 6-well plates. Following 72 h of growing in co-culture, the cells were treated with 250 nM adr, 50 nM doc and PBS (vehicle) for 24 h. Residual cell number was estimated using traditional counting methods, and the percentage of GFP-S was measured by fluorescence-activated cell sorting (FACS). Number of residual GFP-S  =  Residual cell number * Percentage of GFP-S. Cell apoptosis was evaluated using the Annexin-V-FITC Apoptosis Detection Kit (BD Biosciences, USA) in accordance with the the manufacturer's instruction. Briefly, both floating and adherent cells were collected, washed twice with cold PBS and resuspended in 1 × binding buffer. A total of 5 µl Annexin-V-FITC and 10 µl propidium iodide were also added, followed by a 15-min incubation at room temperature in the dark. The Annexin-V-FITC binding on GFP-S was analyzed by flow cytometry (BD FACSCalibur, USA) using FITC signal detector (excitation: 488 nm; emission: 530 nm).

Effects of exosomes on cell cycle regulation were studied on MCF-7/S (1×10^5^ cells per well) seeded in 6-well plates. After cells had attached, the media were removed and fresh media containing vehicle, 20 µg/ml A/exo, RNase A/exo, D/exo, RNase D/exo, S/exo, and RNase S/exo were added. Following a 72-hour incubation, single cell suspension of MCF-7/S was fixed in 1 ml precooling 75% alcohol at −20°C over night, washed with PBS, and treated with propidium iodide and RNase at 37°C for 30 min (avoiding light). Then the G0/G1, S, and G2/M phase fractions were determined by flow cytometry (BD FACSCalibur, USA). Apoptosis assay was carried out on MCF-7/S pretreated for 72 h at 37°C with vehicle, A/exo, RNase A/exo, D/exo, RNase D/exo, S/exo, and RNase S/exo. After 24 h exposure to 250 nM adr or 50 nM doc, apoptotic rate of MCF-7/S was analyzed as described above.

### RNA extraction and microarray

RNA was extracted from different exosomes and cell preparations using the Total Exosome RNA and Protein Isolation Kit (Invitrogen, USA) and mirVana RNA Isolation Kit (Ambion, USA) according to the manufacturer's protocols. RNA was quantified spectrophotometrically (Thermo Scientific, USA), and its quality was assessed by an Agilent 2100 Bioanalyzer (Agilent Technologies, USA).

Microarray was done to characterize the miRNA expression profiles of A/exo, D/exo and S/exo. According to Affymetrix manufacturer's recommendation, exosomal RNA was labeled using the Flash Tag RNA Labeling Kit (Genisphere, USA). Hybridization and washing were then performed via the Affymetrix Fluidics Station 450 and Hybridization Oven 640 under standard conditions. Image processing was conducted using the Affymetrix Gene Array 3000 scanner, and raw data files of each sample were treated with the Affymetrix miRNA QC Tool software. The Affymetrix Gene Chip miRNA 3.0 Array contains 19,724 probe sets including 1,733 human mature miRNAs. Differentially expressed miRNAs were filtered to exclude those changes less than 2.0-fold compared with S/exo.

### Quantitative real-time polymerase chain reaction (qRT-PCR)

To validate some miRNAs detected by microarray, qRT-PCR, applying SYBR green technique, was carried out. Briefly, total RNA from harvested exosomes was extracted as described above. cDNA for miRNA was synthesized using the BU-Script RT Kit (Biouniquer Technology, China) on an iCycler iQ system (Bio-Rad, USA). Five pairs of miRNA specific primers were used for PCR, and *U6* was used as an internal control (Table 1). SYBR green qRT-PCR amplifications were performed on a Light Cycler 480 (Roche, Australia). The thermal profile for qRT-PCR was 91°C for 5 min followed by 45 cycles of 91°C for 15 sec, 60°C for 30 sec, followed by melting curve detection. The Ct values for each miRNA were normalized to *U6*, and the relative expressions were calculated using the ΔΔCt method.

**Table 1:** Primers used for qRT-PCR validation.

miRNA	Forward Primer (5′-3′)	Reverse Primer (5′-3′)
miR-100	GAACCCGTAGATCCGAACT	CAGTGCGTGTCGTGGAGT
miR-17	GCAAAGTGCTTACAGTGCAG	CAGTGCGTGTCGTGGAGT
miR-222	GCGAGCTACATCTGGCTACT	CAGTGCGTGTCGTGGAGT
miR-342-3p	GCCTCTCACACAGAAATCG	CAGTGCGTGTCGTGGAGT
miR-451	AAACCGTTACCATTACTGAG	CAGTGCGTGTCGTGGAGT
miR-30a	GCGCTGTAAACATCCT	CAGTGCGTGTCGTGGAGT
U6	CGCAAGGATGACACG	GAGCAGGCTGGAGAA

To determine whether exosomes could alter the gene expressions in recipient cells, 20 µg/ml A/exo or D/exo was co-cultured with MCF-7/S for 72 h in 6-well plates as described above. These samples were designated as MCF-7/S + A/exo and MCF-7/S + D/exo for simplicity and were referred to as the “acquired” cells. In selected experiments, *miR-222* mimics (15 nM) or control mimics were transfected into MCF-7/S (MCF-7/S + *miR-222* mimics and MCF-7/S + control mimics) using Lipofectamine 2000 (Invitrogen, USA) according to the manufacturer's instruction. At 24 h after transfection, the cells were reseeded in 6-well plates for another 48 h. Then, total RNA was extracted and pooled as per manufacturer's protocols from i) MCF-7/S, ii) MCF-7/S + A/exo or MCF-7/S + D/exo, iii) MCF-7/Adr or MCF-7/Doc, iv) A/exo or D/exo, v) MCF-7/S + *miR-222* mimics, and vi) MCF-7/S + control mimics. As for quantification of miRNA and mRNA, cDNA synthesis and PCR analysis were performed using the BU-Script RT Kit (Biouniquer Technology, China) with SYBR green. Expressions of phosphatase and tensin homolog (*PTEN*) mRNA and β-actin internal control were performed using following primers: *PTEN* forward, 5′-TGGCGGAACTTGCAATCCTCAGT-3′, and reverse, 5′-TCCCGTCGTGTGGGTCCTGA-3′; β-actin forward, 5′-CACCTTCTACAATGAGCTGCGTGTG-3′, and reverse, 5′-ATAGCACAGCCTGGATAGCAACGTAC-3′. All of the reactions, including the negative control (nuclease-free water as a template), were run in triplicate.

### Target gene analysis

Three softwares PicTar (http://pictar.mdc-berlin.de/), TargetScan (http://www.targetscan.org/), and MicroCosm (http://www.ebi.ac.uk/enright-srv/microcosm/htdocs/targets/v5/) were employed to predict miRNA targets. The predicted genes of individual miRNA were uploaded to the online DAVID program (http://david.abcc.ncifcrf.gov/) for their functional annotation clustering analysis. The predominant biological pathways for the selected miRNAs were identified.

### Statistical methods

Statistical analysis was performed using the SPSS 16.0 package. All experiments were carried out in triplicate, and the representative data were presented. Differences were determined by Student's t test or by ANOVA followed by the Newman-Keuls multicomparison test when appropriate. A value of *P*<0.05 was considered significant.

## Results

### Resistance transmission in co-cultures

**Figure 1:**
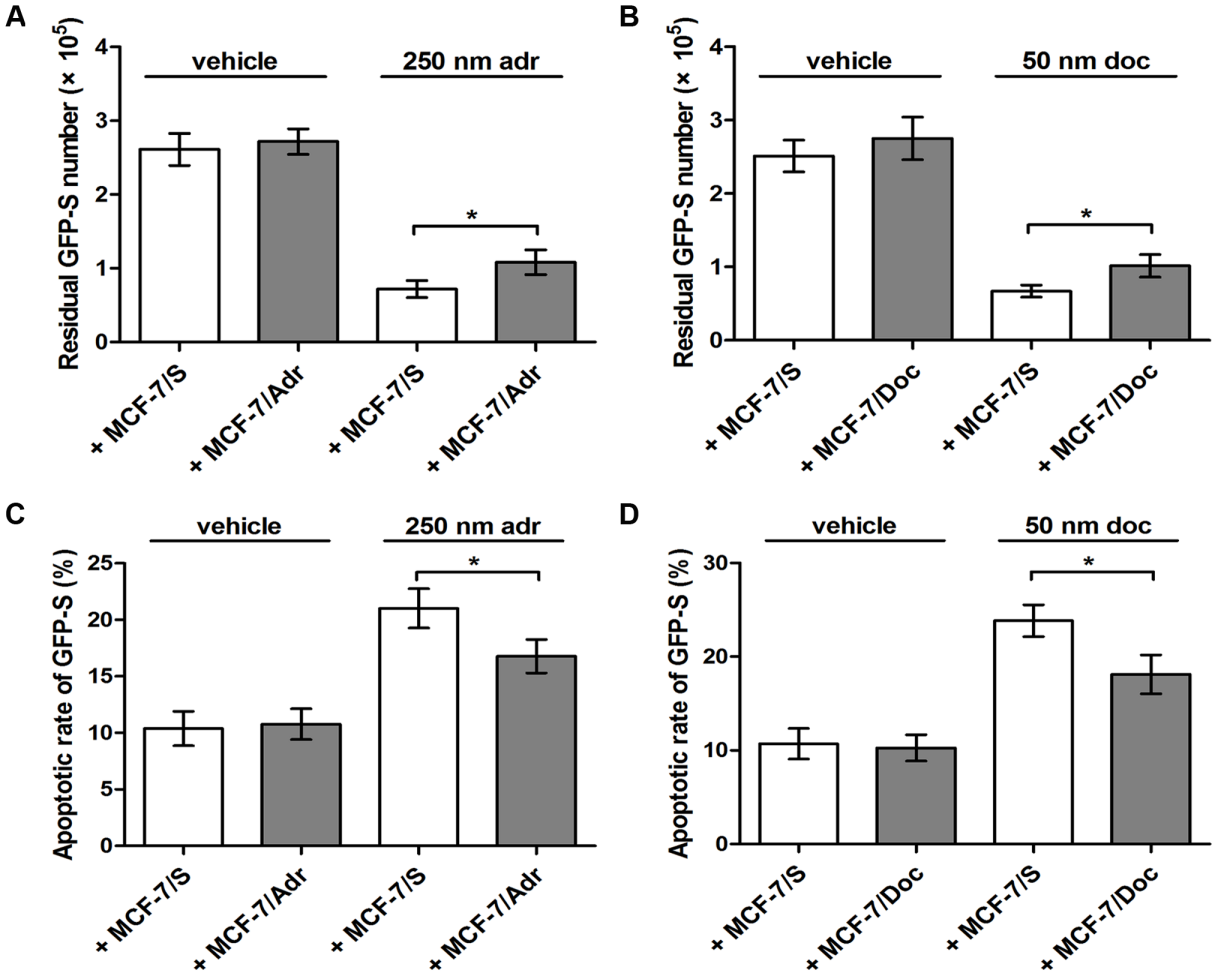
Effects of cell co-cultures. (A) Number of residual GFP-S was evaluated after cell mixture was treated with vehicle or 250 nm adr for 24 h. (B) Number of residual GFP-S was evaluated after cell mixture was treated with vehicle or 50 nm doc for 24 h. (C) Apoptotic rate of GFP-S was determined after cell mixture was treated with vehicle or 250 nm adr for 24 h. (D) Apoptotic rate of GFP-S was determined after cell mixture was treated with vehicle or 50 nm doc for 24 h. Data are expressed as the mean ± SD, n = 3: * *P*<0.05, + MCF-7/Adr vs. + MCF-7/S or + MCF-7/Doc vs. + MCF-7/S.

Since the intercellular transmission of drug resistance occurred between different tumor cell types and led to increased survival potential [13], we aimed to test whether MCF-7/Adr and MCF-7/Doc were able to alter the chemoresponse in MCF-7/S. To be easily observed, MCF-7/S was transfected before seeding with the lentivirus encoding green fluorescent protein (and, therefore, called GFP-S). GFP-S and daughter GFP-S with inherited green fluorescence could be clearly identified in co-cultures over several days. Then they were maintained in a 1∶1 ratio, for 72 h, under three conditions: i) with the MCF-7/Adr selected at 500 nM adr, ii) with the MCF-7/Doc selected at 100 nM doc and iii) with the MCF-7/S grew synchronously in drug-free medium, after which resistance status was assessed in the presence of 250 nM adr, 50 nM doc, and vehicle. As seen in Figure 1, treatment of cell mixture with adr or doc reduced GFP-S number in respect to control cells treated with vehicle alone. In addition, co-culture of GFP-S cells and MCF-7/Adr or MCF-7/Doc resulted in significantly more surviving GFP-S after drug exposure, as compared to those mixed with MCF-7/S (Fig.1A and B; * *P*<0.05). Consistent with an increase of the residual GFP-S number, we showed by apoptosis assay that incubation of GFP-S with MCF-7/Adr or MCF-7/Doc significantly inhibited apoptosis induced by toxic insult (Fig.1C and D; * *P*<0.05). These suggested that MCF-7/Adr and MCF-7/Doc, but not MCF-7/S, could potentially spread chemoresistance to recipient cells.

### Characterization of exosomes

**Figure 2:**
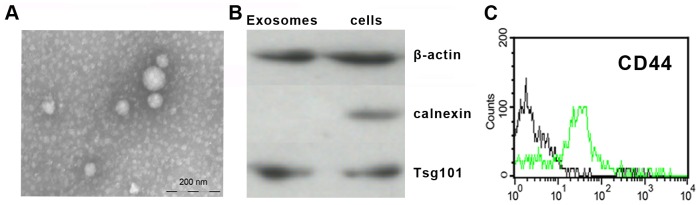
Characterization of exosomes. (A) Representative micrograph of transmission electron microscopy of D/exo showing a spheroid shape. Bar indicates 200 nm. (B) Western blot for the exosome-related proteins Tsg101 and β-actin and endoplasmic reticulum protein calnexin in D/exo and MCF-7/Doc. D/exo is positive for Tsg101 and β-actin, while no calnexin is detected. (C) Representative cytofluorimetric analysis of D/exo showing the expression of CD44 (green line) surface molecule. Black line represents the isotypic control. For A, B and C, exosome preparations of 3 A/exo, 3 D/exo, and 3 S/exo were analyzed with similar results.

Mounting evidence indicates that tumor-derived exosomes play an important role in therapy failure [2]. To investigate their correlation with resistance transmission, we first collected A/exo, D/exo, and S/exo from supernatants of MCF-7/Adr, MCF-7/Doc, and MCF-7/S cells through a series of centrifugation and ultracentrifugation steps. The characteristics of exosomes were determined by electron microscopy, Western blot and flow cytometry. Transmission electron microscopy showed that the exosomes were homogeneous in morphology and ranged from 50 to 100 nm in size (Fig.2A). Western blotting for the exosome-related proteins Tsg101 and β-actin and endoplasmic reticulum protein calnexin revealed that the exosomes were positive for Tsg101 and β-actin, while calnexin was not detected (Fig.2B). Besides, by FACS analysis, all exosomes expressed surface molecule CD44 (Fig.2C). Together, these verified that the examined vesicles truly were exosomes and that A/exo, D/exo and S/exo could be isolated in a consistent manner.

### Uptake of exosomes

**Figure 3:**
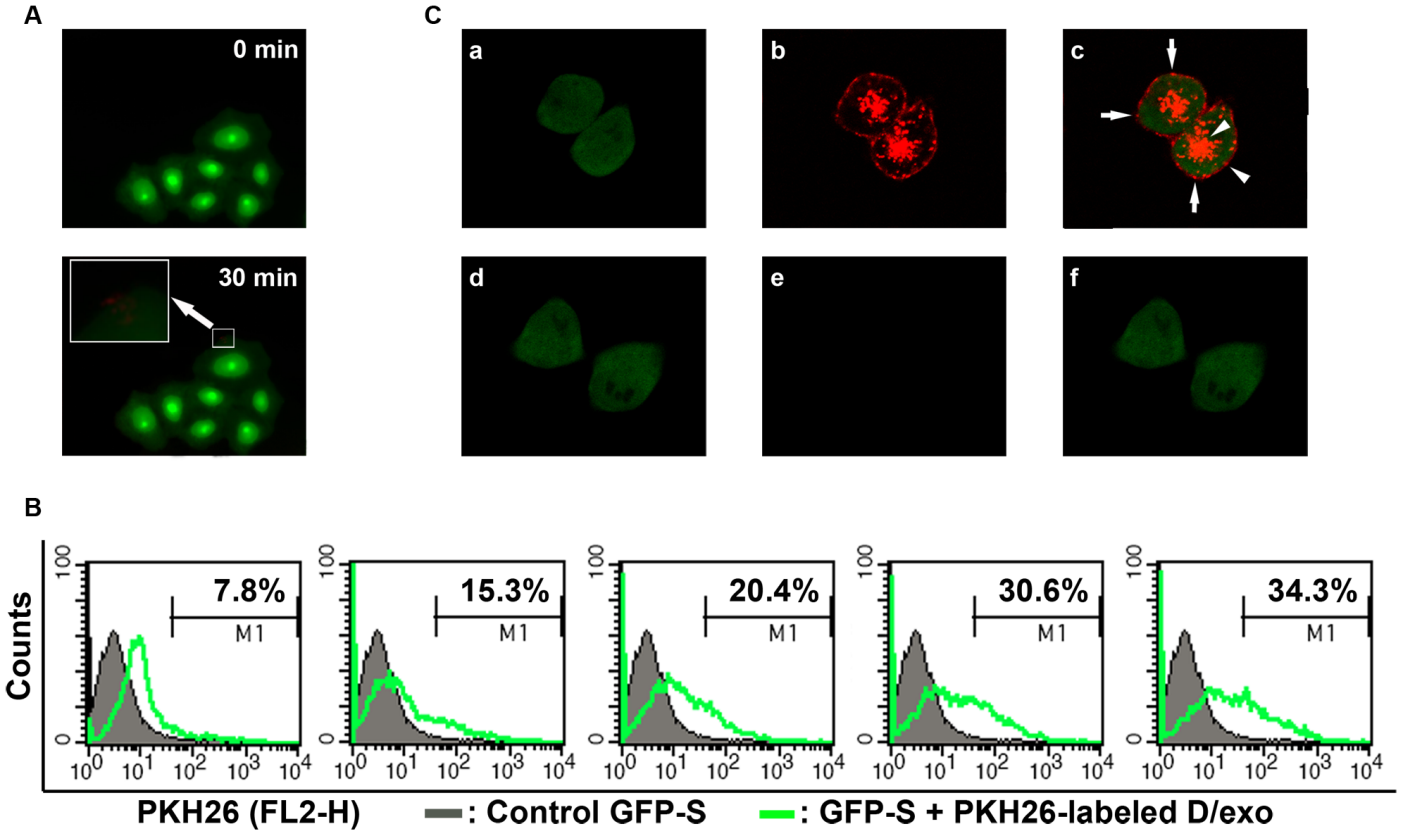
Uptake of D/exo into recipient cells. (A) Representative fluorescence microscopy of GFP-S exposed to PKH26-labeled D/exo for 0 min and 30 min. (B) Representative flow cytometry of GFP-S incubated with PKH26-labeled D/exo for 30 min, 60 min, 90 min, 120 min, and 150 min. Nearly 7.8%, 15.3%, 20.4%, 30.6%, and 34.3% of GFP-S showed PKH26 fluorescence (green line) with respect to the cells treated with unlabeled exosomes (black line). (C) Representative confocal microscopy of GFP-S exposed to PKH26-labeled D/exo for 24 h. (a) Green signal from GFP-S. (b) Red signal from PKH26-labeled D/exo. (c) Overlay of a and b. Arrows indicate punctiform signal, probably standing for docked D/exo. Arrowheads represent diffuse signal, likely from the internalization and diffusion of PKH26. (d) Green signal from control GFP-S with unlabeled D/exo. (e) No red signal can be detected in unlabeled D/exo. (f) Merging of d and e. Three experiments were carried out with similar results.

Recent studies demonstrated the transfer of exosomes from donor cells to recipient cells. Then we asked whether A/exo and D/exo could adhere to MCF-7/S, i.e. are present on their surface, or are also engulfed, i.e. localized intracellularly. To better observe the interaction, GFP-S was employed and D/exo was stained with PKH26 dye. When PKH26-labeled D/exo was added to GFP-S for 30 min, red fluorescence on the cell membrane was detected in GFP-S by fluorescence microscopy, suggesting the binding and incorporation of D/exo to the cell surface (Fig.3A). To establish a dynamic process, GFP-S was treated with 20 µg/ml PKH26-labeled D/exo at 37°C for 30 min, 60 min, 90 min, 120 min, and 150 min. Flow cytometry analysis for the resultant recipient GFP-S displayed a 7.8%, 15.3%, 20.4%, 30.6%, and 34.3% increase in PKH26 labeling compared to the cells alone, respectively (Fig.3B). The quantitative flow cytometry data, on continuous absorption, were further supported by the images from confocal laser scanning microscopy. After a 24-hour incubation, multiple red vesicles could be seen docked on GFP-S and most diffuse PKH26 signals were concentrated in the cytoplasm (Fig.3C). Moreover, the uptake of exosomes was highly efficient as all GFP-S cells were positive for stained D/exo. The negative control, GFP-S incubated with unlabeled D/exo, did not show any red fluorescence when visualized with the PKH26 filter set (Fig.3C). Similar results were obtained with respect to A/exo (not shown). Hence, A/exo and D/exo not only adhered to MCF-7/S but were also engulfed and internalized.

### Effects of A/exo and D/exo

**Figure 4:**
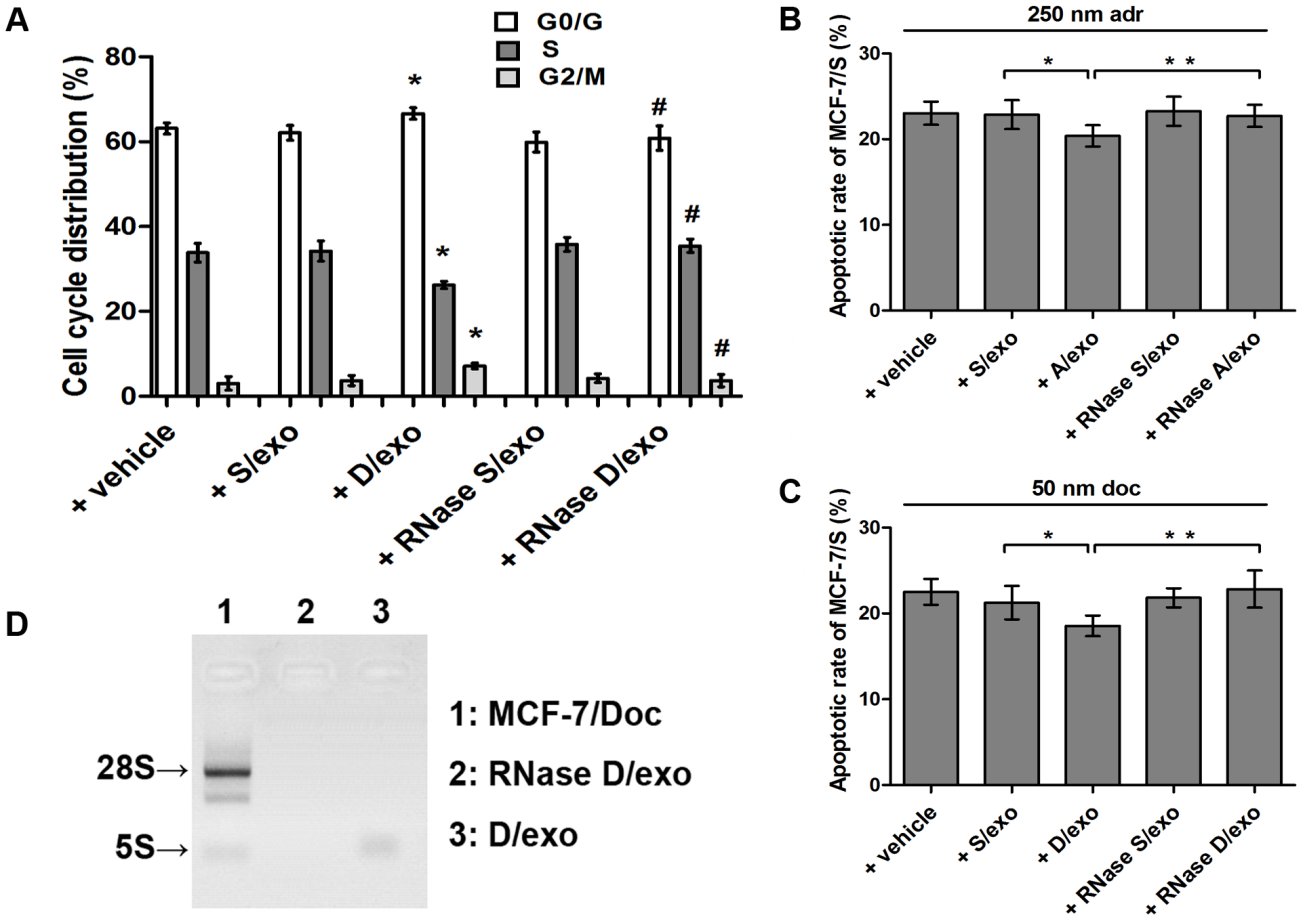
Effects of different exosomes. (A) Flow cytometry of cell cycle distribution was analyzed after MCF-7/S was incubated with vehicle, S/exo, D/exo, RNase S/exo, and RNase D/exo for 72 h. Data are expressed as the mean ± SD, n = 3: * *P*<0.05, + D/exo vs. + S/exo; # *P*<0.05, + RNase D/exo vs. + D/exo. (B) Evaluation of MCF-7/S incubated with vehicle, S/exo, A/exo, RNase S/exo, and RNase A/exo for 72 h. Apoptotic rate of MCF-7/S was then determined after 24 h exposure to 250 nM adr. Data are expressed as the mean ± SD, n = 3: * *P*<0.05, + A/exo vs. + S/exo; ** *P*<0.05, + RNase A/exo vs. + A/exo. (C) Evaluation of MCF-7/S incubated with vehicle, S/exo, D/exo, RNase S/exo, and RNase D/exo for 72 h. Apoptotic rate of MCF-7/S was then determined after 24 h exposure to 50 nM doc. Data are expressed as the mean ± SD, n = 3: * *P*<0.05, + D/exo vs. + S/exo; ** *P*<0.05, + RNase D/exo vs. + D/exo. (D) D/exo treated with RNase showed a significant difference in RNA degradation compared to control exosomes.

To examine whether A/exo and D/exo could be responsible for spreading chemoresistance, we next compared their effects with S/exo. We first evaluated the uptake of D/exo and S/exo stained with PKH26 dye by GFP-S, after a 24-hour incubation at 37°C. Confocal microscopic observations showed a similar co-localization of the PKH26-labeled exosomes and recipient cells, suggesting that GFP-S absorbed in equal manner both D/exo and S/exo (not shown). Transmitted chemoresistance was subsequently assessed in exosome-treated MCF-7/S by analyzing cell cycle distribution and by determining apoptotic rates in the presence of 50 nM doc. As seen in Figure 4, incubation of MCF-7/S cells with 20 µg/ml D/exo increased G1/G2 phase and decreased S phase in respect to control cells incubated with S/exo (Fig.4A; * *P*<0.05). Furthermore, the apoptotic rate induced by 50 nM doc was relatively greater in MCF-7/S added with S/exo, whereas a marked reduction in apoptotic rate was found in MCF-7/S pretreated with D/exo (Fig.4C; * *P*<0.05). Likewise, A/exo, but not S/exo, significantly modulated the cell cycle distribution (not shown) and adr-induced apoptosis (Fig.4B; * *P*<0.05), with respect to S/exo. These data were in good agreement with the preceding co-culture experiment, further indicating that such effects should be ascribed to exosomes shed by drug-resistance cells.

When A/exo and D/exo were treated with high, unphysiological concentration of RNase, that was shown to trigger a complete degradation of the vesicle-contained RNA without affecting their structure [22], the effects on increased G1/G2 phase (Fig.4A; # *P*<0.05) and apoptosis resistance (Fig.4B and C; ** *P*<0.05) of MCF-7/S were remarkably impaired. We also showed by electrophoresis that RNase treatment significantly degraded the RNA carried by D/exo (Fig.4D). Thus, it was revealed that the biological functions of A/exo and D/exo on MCF-7/S were mediated by the shuttle of RNA following exosome internalization.

### Detection and profiling of exosomal RNA

**Figure 5:**
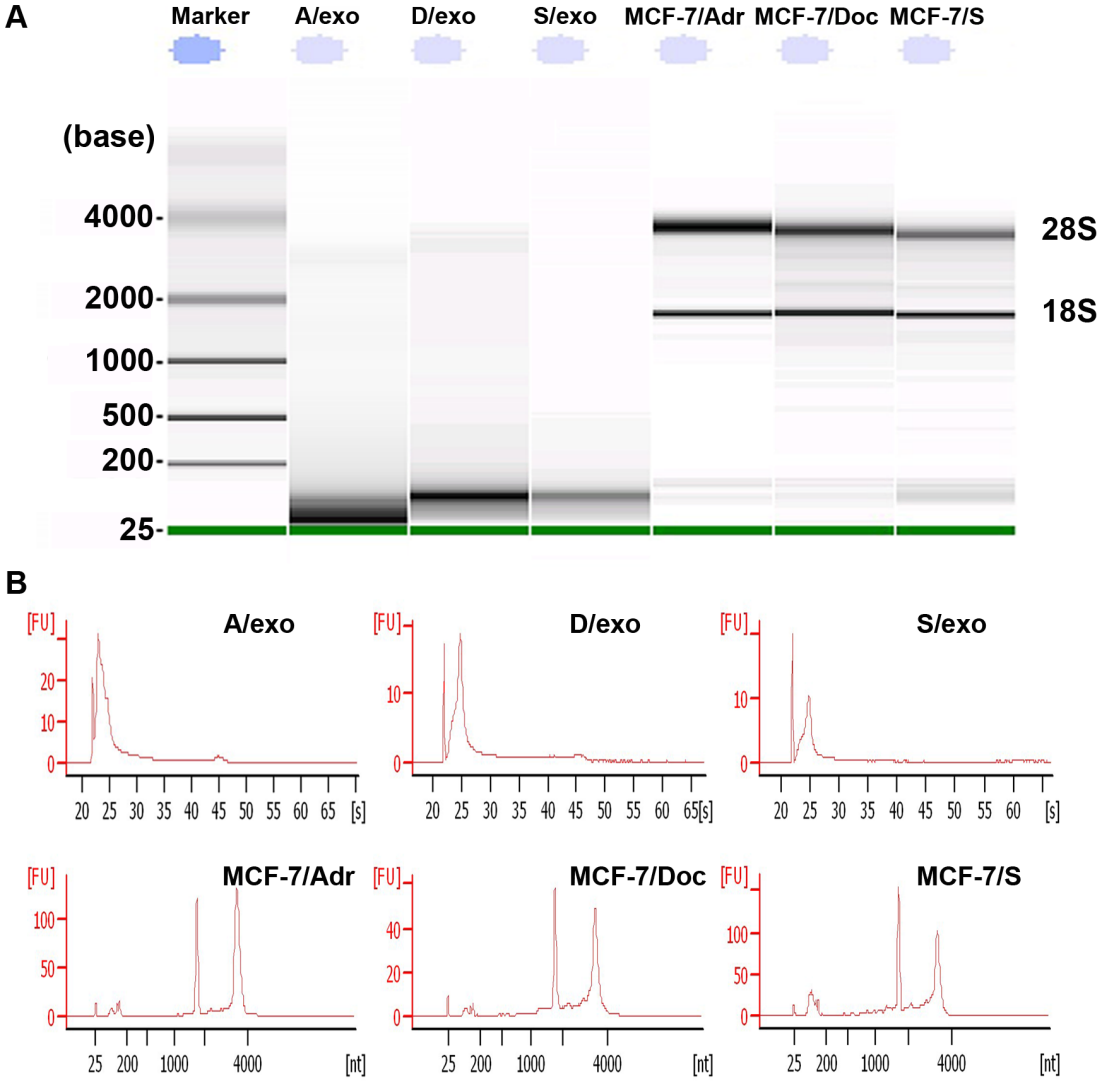
Detection of exosomal RNA. (A) RNA content is remarkably different, with the majority of exosomal RNA below 200 bases in size and with little or no bands of 28S and 18S ribosomal RNA, compared to RNA from donor cells. (B) Representative bioanalyzer profile of the RNA contained in A/exo, D/exo, S/exo and their cells of origin showing that the 28S and 18S ribosomal RNA were absent or barely detectable in exosomal RNA. Three different samples tested in triplicate were analyzed with similar results.

To confirm whether RNA truly existed in three exosome samples, we extracted total RNA from A/exo, D/exo, S/exo and their cells of origin. The exosomal RNA showed little or no bands of 28S and 18S ribosomal RNA, whereas an obvious band was detected below 200 bases (Fig.5A). These, along with the relevant peak characteristic of small RNA classes separated by bioanalyzer (Fig.5B), strongly indicated that exosomal RNA was heterogeneous and, more importantly, was enriched for small RNA of the size of miRNAs.

**Figure 6:**
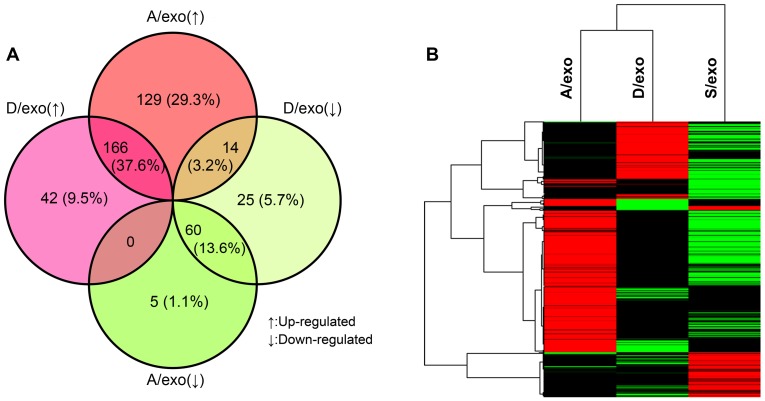
Profiling of exosomal RNA. (A) The percentage distribution of the 441 differentially expressed miRNAs in A/exo and D/exo compared with S/exo. (B) Hierarchical cluster analysis of miRNA expression profiles in A/exo and D/exo compared with S/exo. Black color stands for a median transcript level. Red color indicates a transcript level above the median level, and green color represents a transcript level below the median level of the particular assay as measured in all samples.

We recently analyzed the miRNA profiles of MCF-7/Adr, MCF-7/Doc and MCF-7/S to explore which miRNAs might be associated with chemoresistance [15]. To test the hypothesis that A/exo, D/exo and S/exo also loaded selective patterns of miRNAs, their miRNA expressions were screened by microarray. Compared with S/exo, there were 441 differentially expressed miRNAs (at least 2.0-fold changes) in A/exo and D/exo. Specifically, while 166 miRNAs were up-regulated, 60 miRNAs were down-regulated in both A/exo and D/exo; 129 miRNAs were increased and 5 miRNAs were decreased only in A/exo; 42 miRNAs were elevated and 25 miRNAs were reduced exclusively in D/exo; 14 miRNAs were up-regulated in A/exo and simultaneously down-regulated in D/exo; and no miRNAs were increased in D/exo but decreased in A/exo (Fig.6A). Then we performed an agglomerative hierarchical clustering using the 441 miRNAs through average linkage. The data shown had been subjected to median normalization of each miRNA across all samples. Each listed miRNA was significantly differentially expressed (at least 2.0-fold changes) in A/exo and/or D/exo compared with S/exo (Fig.6B).

**Figure 7:**
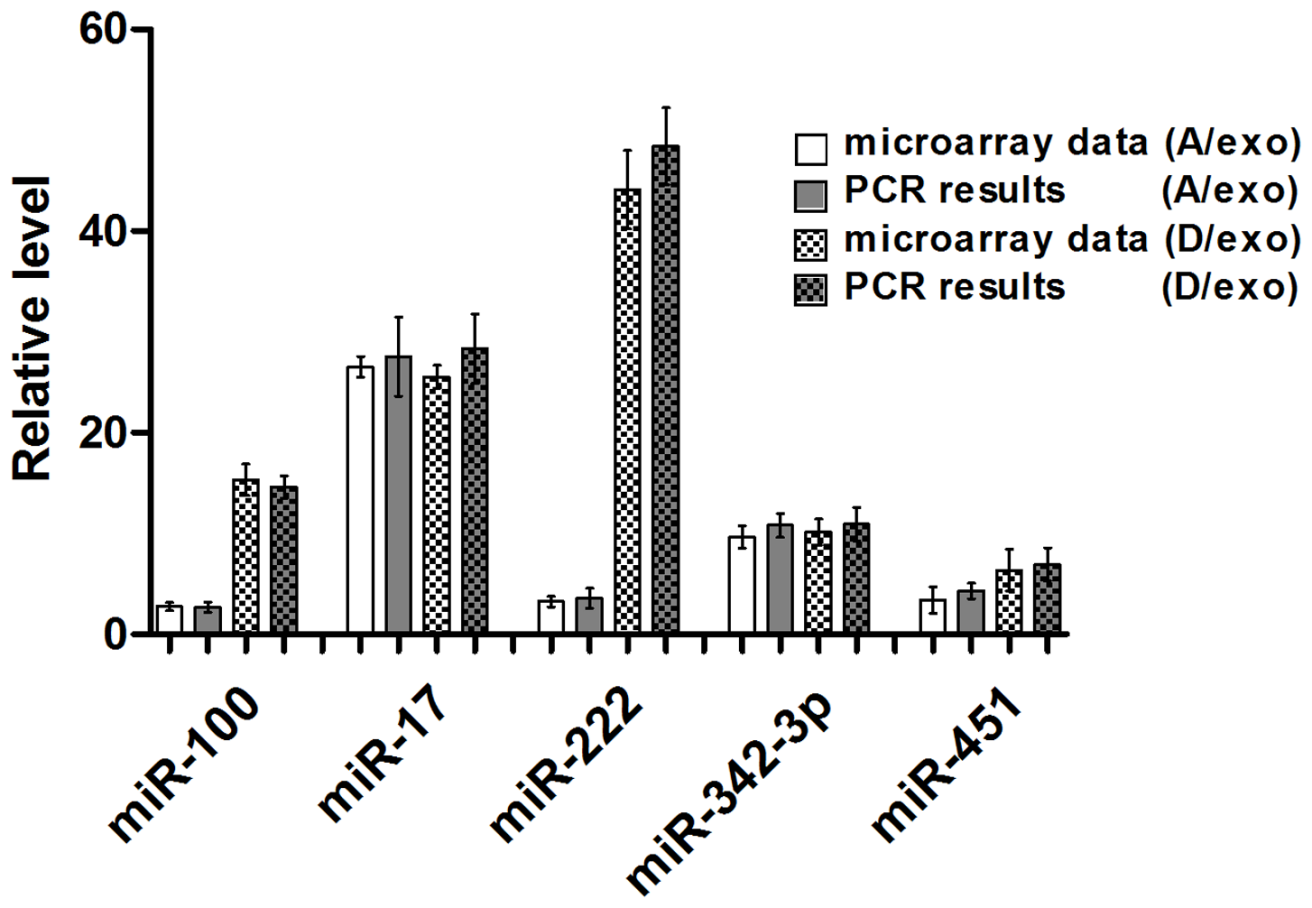
Validation for microarray. qRT-PCR validation of the microarray data of the five miRNAs with consistent expression changes in A/exo and D/exo. Compared to S/exo, the levels of *miR-100*, *miR-17*, *miR-222*, *miR-342-3p* and *miR-451* were significantly up-regulated in A/exo and D/exo using qRT-PCR. Data are normalized to *U6*. Fold changes from microarray and qRT-PCR are expressed as the mean ± SD, n = 3.

We next carried out qRT-PCR to assess the data obtained from microarray. Five miRNAs were selected for validation, all of which (*miR-100*, *miR-17*, *miR-222*, *miR-342-3p* and *miR-451*) were elevated in both A/exo and D/exo. In all cases, PCR results showed good consistency with microarray data (Fig.7), revealing that the latter accurately reflected miRNA expression differences among A/exo, D/exo, and S/exo.

### Exosomes transfer miRNAs to recipient cells

**Figure 8:**
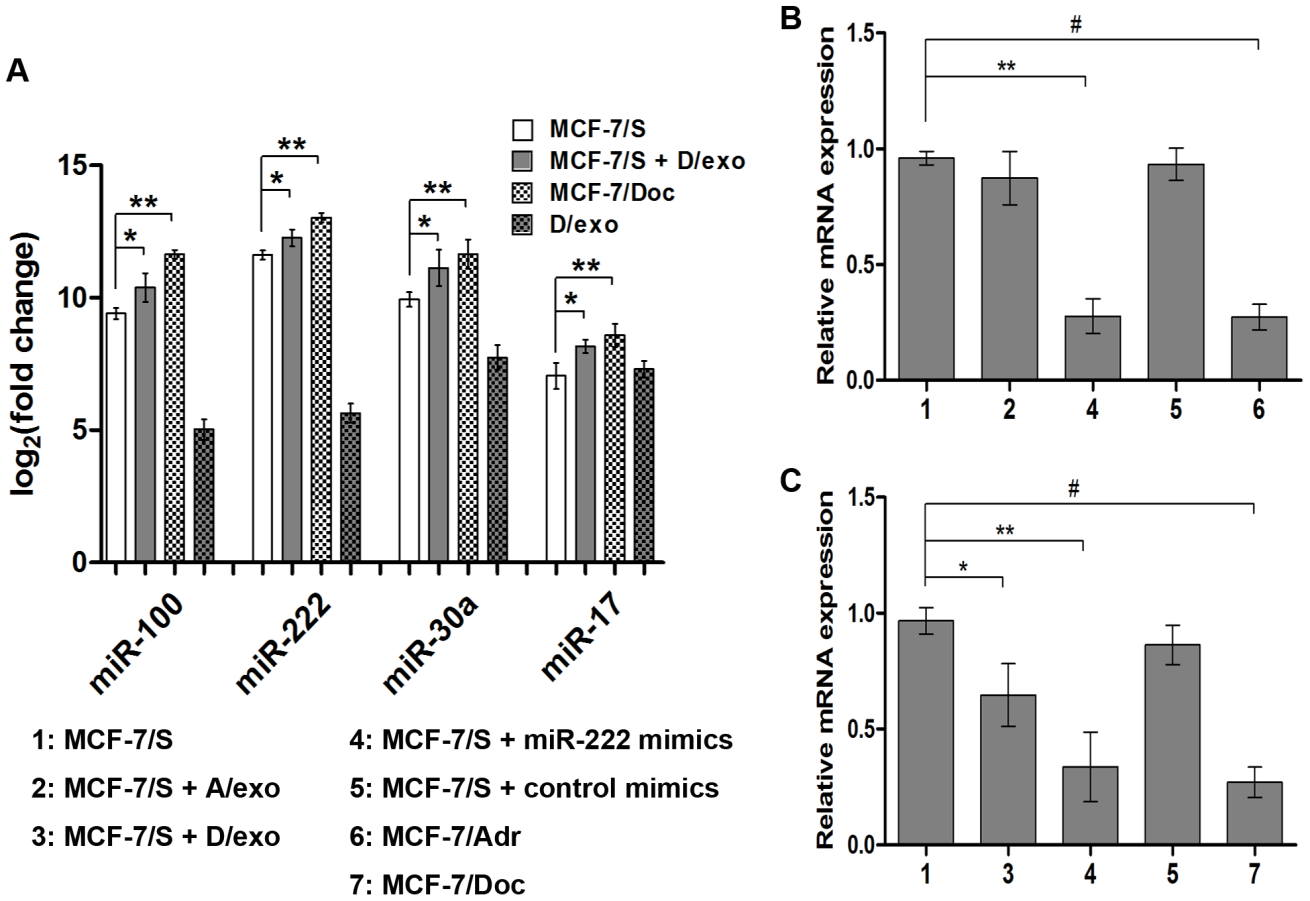
Transfer of miRNAs and gene regulation. (A) *miR-100*, *miR-222*, *miR-30a* and *miR-17* were analyzed in D/exo, MCF-7/Doc, the recipient MCF-7/S before and after D/exo incubation. Data are expressed as the mean ± SD, n = 3: * *P*<0.05, MCF-7/S vs. MCF-7/S + D/exo; ** *P*<0.05, MCF-7/S vs. MCF-7/Doc. (B) Relative expression of *PTEN* in MCF-7/S, MCF-7/Adr, MCF-7/S treated with A/exo and transfected with *miR-222* mimics. Data are expressed as the mean ± SD, n = 3: ** *P*<0.05, MCF-7/S vs. MCF-7/S + *miR-222* mimics; # *P*<0.05, MCF-7/S vs. MCF-7/Adr. (C) Relative expression of *PTEN* in MCF-7/S, MCF-7/Doc, MCF-7/S treated with D/exo and transfected with *miR-222* mimics. Data are expressed as the mean ± SD, n = 3: * *P*<0.05, MCF-7/S vs. MCF-7/S + D/exo; ** *P*<0.05, MCF-7/S vs. MCF-7/S + *miR-222* mimics; # *P*<0.05, MCF-7/S vs. MCF-7/Doc.

The presence of selective miRNA patterns in A/exo and D/exo opens up the intriguing possibility that these miRNAs may be transferred by exosomes. We tested this hypothesis by incubating D/exo with MCF-7/S and compared the levels of miRNAs found in MCF-7/S, MCF-7/Doc, and D/exo with those of acquired cells. qRT-PCR analysis showed that both MCF-7/S and MCF-7/Doc as well as D/exo carried *miR-100*, *miR-222*, *miR-30a* and *miR-17* (Fig.8A; ** *P*<0.05). Moreover, these miRNAs were present at significantly higher levels in acquired cells relative to the control MCF-7/S (Fig.8A; * *P*<0.05), suggesting transfer. Likewise, the co-culture of MCF-7/S with A/exo resulted in significantly fold higher expressions of *miR-100*, *miR-222*, *miR-30a* and *miR-17*, as compared to MCF-7/S (not shown).

### Target gene prediction and pathway mapping analysis

To evaluate the functions of exosome-loaded miRNAs, we further checked their potential targets by three computational algorithms: PicTar [23], TargetScan [24], and MicroCosm [25]. The genes detected by at least two of three independent methods were considered to be the targets of miRNAs. Here, we chose three miRNAs (*miR-100*, *miR-222* and *miR-30a*) with the significantly increased expression in both A/exo and D/exo for prediction because all these miRNAs displayed a consistent up-regulation in both MCF-7/Adr and MCF-7/Doc as we previously reported [15]. Then we assigned the putative targets into KEGG pathway enrichment analysis using a web-based tool DAVID [26]. As shown in Table 2, there was a broad range of biological pathways, such as “pathways in cancer”, “MAPK signaling pathway”, “cell cycle”, “axon guidance”, and “calcium signaling pathway”. Of these significant pathways identified for the miRNAs, one was related to exosome release (“calcium signaling pathway”) and four to drug resistance (“pathways in cancer”, “MAPK signaling pathway”, “cell cycle” and “axon guidance”). To preliminarily assess the contribution of exosome-contained miRNAs, we chose *miR-222* and its target gene *PTEN*. qRT-PCR showed that MCF-7/S expressed *PTEN* intrinsically, whereas MCF-7/Adr and MCF-7/Doc displayed a marked reduction of *PTEN* (Fig.8B and C; # *P*<0.05). Additionally, *PTEN* in MCF-7/S transfected with *miR-222* mimics was strikingly decreased compared with that in control MCF-7/S, suggesting that *miR-222* could regulate drug resistance by targeting *PTEN* (Fig.8C; ** *P*<0.05). This reduced expression of *PTEN* was also revealed when adding D/exo to MCF-7/S (Fig.8C; * *P*<0.05). Although *PTEN* was present at lower level in *miR-222* mimics-treated MCF-7/S relative to MCF-7/S (Fig.8B; ** *P*<0.05), its expression did not show a trend for lower expression in MCF-7/S receiving A/exo.

**Table 2:** KEGG pathway enrichment analysis with DAVID tool.

miRNA	Term	Gene Count (%)	*P*-Value	Genes
miR-100	hsa05200:Pathways in cancer	3(25.00)	0.023	FRAP1,FGFR3,FZD8
miR-222	hsa05200:Pathways in cancer	5(7.25)	0.013	ETS1,ARNT,CDKN1B,FOS,MAPK10
	hsa04010:MAPK signaling pathway	4(5.80)	0.041	NTF3,PPP3R1,FOS,MAPK10
	hsa04722:Neurotrophin signaling pathway	3(4.35)	0.050	YWHAG,NTF3,MAPK10
	hsa04110:Cell cycle	3(4.35)	0.051	CDKN1C,CDKN1B,YWHAG
	hsa04360:Axon guidance	3(4.35)	0.054	GNAI2,PPP3R1,SEMA6D
miR-30a	hsa05414:Dilated cardiomyopathy	7(3.14)	0.002	CACNB2,ADRB1,ITGB3,DMD,SGCB,ATP2A2,CACNA1C
	hsa04020:Calcium signaling pathway	9(4.04)	0.003	GRM5,ADRB2,CAMK2D,ADRB1,PPID,PLCB4,PLCG1,ATP2A2,CACNA1C
	hsa05412:Arrhythmogenic right ventricular cardiomyopathy (ARVC)	6(2.69)	0.004	CACNB2,ITGB3,DMD,SGCB,ATP2A2,CACNA1C
	hsa05410:Hypertrophic cardiomyopathy	6(2.69)	0.007	CACNB2,ITGB3,DMD,SGCB,ATP2A2,CACNA1C
	hsa04722:Neurotrophin signaling pathway	7(3.14)	0.008	RAP1B,IRS1,CAMK2D,CRKL,YWHAZ,PLCG1,MAP3K5
	hsa04720:Long-term potentiation	5(2.24)	0.016	RAP1B,GRM5,CAMK2D,PLCB4,CACNA1C
	hsa04730:Long-term depression	5(2.24)	0.017	LYN,GRM5,GNAI2,PLCB4,GNAO1
	hsa04930:Type II diabetes mellitus	4(1.79)	0.029	IRS1,SOCS3,SOCS1,CACNA1C
	hsa04360:Axon guidance	6(2.69)	0.037	GNAI2,UNC5D,PLXNA1,EPHB2,SEMA6D,SEMA3A
	hsa04120:Ubiquitin mediated proteolysis	6(2.69)	0.046	CUL2,NEDD4,UBE2I,SOCS3,NEDD4L,SOCS1
	hsa04010:MAPK signaling pathway	8(3.59)	0.087	RAP1B,IL1A,CACNB2,MAP3K12,CRKL,RRAS2,CACNA1C,MAP3K5

## Discussion

Chemoresistance is a major stumbling block to the successful treatment of BCa as tumor cells either fail to reduce in size after toxic insult or the cancer recurs subsequent to an initial “positive” response [1]. Better understanding of the resistance mechanisms is urgently needed in order to improve the clinical efficiency of current regimens. Since adr and doc are two agents commonly used in BCa patients, MCF-7/Adr, MCF-7/Doc, and the paired sensitive cells MCF-7/S were therefore chosen as the subjects in this study.

Levchenko et al. recently demonstrated that several parental cell lines mixed with an equivalent amount of their drug-insensitive variants were able to acquire poor chemoresponse and lower rates of proliferation [13]. Here, the transmitted resistance after co-culture was studied by using, as receptors, fluorescent MCF-7/S obtained by transfection with the lentivirus and therefore called GFP-S. In our experiment, both MCF-7/Adr and MCF-7/Doc conferred increased residual GFP-S number and reduced therapy-induced apoptosis when compared with MCF-7/S. As a matter of fact, clone heterogeneity of tumors often results in the coexistence of several cell subpopulations exhibiting different drug susceptibility [12]. Conventional antitumor agents generally eliminate a few BCa cells, leaving insensitive subpopulations undamaged, which, in turn, would potentially spread resistance traits to residual or newly produced BCa cells. Thus, our results about the communication within BCa cell subpopulations would lend support to a putative mechanism whereby resistant cells provide the sensitive ones a survival advantage to adapt to extreme agent exposure.

Classic cell-to-cell communication is mainly regulated by specific junctions [27], direct adhesions [28], and soluble growth factors [29]. Findings over the past few years, however, suggest the existence of an additional form: exosomes. Although no known exosome-specific markers are currently available, they can be detected based on their size and presence/absence of several proteins. In the present work, calnexin, which belongs to endoplasmic reticulum proteins that are not supposed to be sorted into exosomes, was absent in three isolated samples. This, along with the presence of Tsg101, β-actin, and CD44, strongly indicates that the studied vesicles are exosomes. Indeed, many other proteins could be used for exosome characterization [30], such as tetraspanins (CD9, CD63 and CD81), heat-shock proteins (Hsp90), cytoskeletal proteins (moesin) and metabolism-related proteins (GAPDH). Because protein features would depend on the cell type and may fluctuate according to physiological changes, further fundamental research is necessary to identify the distinctive markers for BCa-derived exosomes. Moreover, given that exosomes may also play a role in “gain and loss” of cell surface receptors [31], it is also important to compare the protein expressions between exosomes and their cells of origin.

Numerous publications have documented the significance of exosomes in intercellular communication and various cells have been shown to communicate via exosomes [3], [32]. Since it was reported that drug-resistant BCa cells are an abundant source of exosomes [18], we examined whether A/exo and D/exo were able to interact with MCF-7/S. Using GFP-S model, the present results showed that D/exo (and A/exo) adhered to and were engulfed by MCF-7/S. This was based on fluorescence microscopy and further confirmed with flow cytometry analysis of GFP-S incubated with PKH26-labeled D/exo (and A/exo). Already, at 30 min 7.8% GFP-S displayed red fluorescence, which was elevated to 34.3% after 150 min indicating that GFP-S incorporated exosomes in a continuous manner. Interestingly, the absorption speed of D/exo (and A/exo) appeared to vary during incubation. The reason for such observations has not been investigated in our study, but, speculatively, the possibility cannot be excluded that either cell recognition molecules on exosome surfaces or specific structures on GFP-S membranes would trigger efficiency in uptake. In addition, albeit speculative, it seems plausible that internalized exosomes might influence the subsequent incorporation. Future research is needed to address the question of whether exosomes are taken up by recipient cells through the process of endocytosis, phagocytosis, direct fusion or ligand-receptor binding [33].

Since exosomes are emerging mediators of intercellular communication, investigations on their relationship to chemotherapy failure are becoming an exciting area. Exosome-mediated resistance transmission has recently been described in several tumor types including ovarian and prostate cancer [16], [17]; however, less is known in BCa. In the present study, we found that MCF-7/S incubated with A/exo (or D/exo) could acquire lower rates of proliferation and higher levels of resistance to a chemotherapeutic drug. Particularly, these properties were ascribed only to A/exo and D/exo, as S/exo was ineffective. Our results are consistent with the preceding co-culture data, raising the possibility that intercellular transfer of exosomes may protect recipient MCF-7/S by both regulating cell cycle distribution and increasing the overall resistance after drug exposure. Under this hypothesis, on one hand, cells that might have been displaced from the tumor due to growth-rate disadvantage could now enter a quiescent stage and be retained; on the other hand, tumor cells would become shielded and chemo-unresponsive due to exosome assistance. We suppose, therefore, that inhibiting transfer of exosomes from drug-resistant BCa cells to sensitive ones could improve efficiency of current regimens. We observed that RNase abrogated the effects of A/exo and D/exo on elevated G1/G2 phase and decreased apoptotic rates in MCF-7/S. Exosomes generally defend RNA from hostile RNase-rich environment [8], but the addition of exosomes with high concentrations of RNase inactivates the RNA. Thus, the significant reduction of exosome biological activities after RNase treatment suggests a putative transfer of RNA from A/exo and D/exo to recipient MCF-7/S. We further confirmed the presence of different subsets of RNA and in particular enrichment for small RNA, including miRNAs. The circulatory miRNAs are being intensively investigated as biomarkers in different cancers [34]. Our observations agree well with previous studies reporting that miRNAs could circulate in various body fluids in a highly stable, cell-free form and that continued secretion of exosomes serves as vehicles for these extracellular miRNAs [35]-[37].

Valadi et al. firstly discovered the exosome-mediated miRNA transfer and promoted the notion that this would be a novel mechanism of genetic exchange between cells [8]. Since then, their seminal finding has generated much enthusiasm in exploring the function of such process during resistance transmission. In the present work, we investigated whether A/exo and D/exo carried selective miRNA patterns that may account for the induction of malignant capacities for MCF-7/S growth and survival. Compared with S/exo, we found 374 differentially expressed miRNAs in A/exo and 307 aberrant miRNAs in D/exo, indicating that exosomes from drug-insensitive BCa cells were characterized by marked changes in miRNA content. At the same time, a large number of the miRNAs exhibited similar expression trends in both A/exo and D/exo, and the others either altered only in certain exosome or fluctuated in opposite directions. Our microarray analysis might suggest the existence of several common pathways as well as drug-specific molecular machineries in spreading resistance traits. Strikingly, while some miRNAs with consistent changes contributed to the cross-resistance, a few miRNAs displayed exclusively in A/exo or D/exo were responsible for the various degree of chemoresponse (own unpublished results). In our opinion, the latter miRNAs are equally noteworthy because comprehensively profiling these miRNAs after using adr and doc, to some extend, would explain the different resistance mechanisms and help to choose an appropriate mono or combined therapeutic program.

The presence of selective miRNA patterns in A/exo and D/exo opens up the intriguing possibility that these miRNAs may be transferred by exosomes. In the present study, both MCF-7/S and resistant sublines as well as their exosomes carried several miRNAs, namely, *miR-100*, *miR-222*, *miR-30a* and *miR-17*. We also demonstrated that following A/exo and D/exo transfer to recipient MCF-7/S, the same miRNAs were significantly increased in acquired cells. Our results would add another piece of evidence to the emerging idea that exosomes from drug-resistant tumor cells are capable of delivering a subset of miRNAs to sensitive cells. In saying this however, we cannot exclude the possibility that increased miRNA levels in acquired cells are caused by either/both direct or indirect exosome-mediated effects on miRNAs. It is therefore desired that further attention be drawn to this field. Interestingly, Yuan et al. found that peak transfer occurred at nearly the same time between 12-36 h and they showed a significant downward trend at 54 h [38]. We have not tested such observation at this time, but we can speculate that the efficiency of miRNA transfer may be different during co-culture.

It is difficult to determine whether all the differentially expressed miRNAs have a major role in the process of resistance transmission. Instead, researchers shift the attention to explore the regulatory capacity of individual miRNAs whose targets are experimentally affirmed. Here we chose *miR-100*, *miR-222* and *miR-30a* for further study because all these miRNAs displayed a consistent up-regulation in A/exo, D/exo, MCF-7/Adr and MCF-7/Doc [15]. After target gene prediction and KEGG pathway analysis, we found one pathway for *miR-100*, five pathways for *miR-222* and eleven pathways for *miR-30a*. In particular, both *miR-100* and *miR-222* were significantly related to “pathways in cancer”, suggesting that increased expression of these two miRNAs may serve as potential biomarkers for BCa. “Cell cycle” regulation is a vital strategy for malignant cells to survive chemotherapy [39]. Our results of increased G1/G2 phase and decreased S phase in MCF-7/S after A/exo or D/exo co-culture might be due to the transfer of *miR-222*. “MAPK signaling pathway” is a well known cascade that accounts for various cellular functions, including proliferation, apoptosis, migration and chemoresistance [40]. “Axon guidance” molecules, which have been well reviewed recently, contribute to BCa initiation and progression, both through autocrine effects on tumor cells as well as paracrine effects on endothelial cells that promote angiogenesis [41]. Then, *miR-222* and its target gene *PTEN* were picked to preliminarily assess the contribution of exosome-contained miRNAs. *PTEN*, one of the most altered tumor suppressor genes in BCa which functions to antagonize the activity of PI3K and block cell proliferation, was inversely correlated with *miR-222* as Garofalo et al. reported [42]. In the present study, we showed that *miR-222*-rich D/exo could alter *PTEN* expression in acquired cells. This was based on *miR-222* mimics transfection experiment and further confirmed by qRT-PCR analysis of *PTEN* within D/exo-treated MCF-7/S. However, this was not observed with A/exo. Perhaps the impaired reduction of *PTEN* in acquired cells was attributed to the low level of *miR-222* observed in A/exo. So far, there is no direct evidence as to the involvement of exosome-delivered *miR-100*, *miR-222* and *miR-30a* in actually modulating the targets within recipient sensitive cells. We are currently examining their actions on several potential resistance-associated proteins as well as signaling pathways. These appear likely because in 2011, Kogure et al. [43] demonstrated that hepatocellular carcinoma-derived exosomes were able to shuttle miRNAs to regulate target gene expression and promote malignant growth in other tumor cells.

## Conclusions

In summary, our study reveals that the ability of drug-resistant BCa cells in transmitting resistance capacity is probably due to their release of exosomes. After binding, absorption, and internalization, these exosomes could alter chemo-susceptibility in recipient sensitive cells by modulating cell cycle distribution and drug-induced apoptosis. Such effects may be partly attributed to the intercellular transfer of specific miRNAs.
